# Supplementary material for: The effects of provisional resin cements on the color and retentive strength of all-ceramic restorations cemented on customized zirconia abutments
Source: PLoS One. 2022 Jan 18;17(1):e0262582. doi: 10.1371/journal.pone.0262582 (PMC8765607; doi:10.1371/journal.pone.0262582)
Supplement: Attachment — Submitted filename: Response to Reviewers.docx [file pone.0262582.s003.docx]

Dear Editor,

Thank you indeed for granting us the chance to promote our findings through your prestigious journal. Appreciations are also expressed to the reviewers for their meticulous review and constructive comments, according to which the manuscript was revised. Following comes a point-by-point response to the reviewers' concerns. Please feel free to contact me for any further queries.

Regards, Dr Rashin Giti

Reviewer #1

1) TempBond is a conventional zinc oxide-eugenol cement and NOT a provisional resin cement. Only two different provisional resin cements (ie, TempBond Clear and Implantlink Semi) were used while TempBond served as control. Please make this clear throughout the whole manuscript.

Response: According to the reviewer's precise comment, the control group was clearly defined throughout the manuscript.

2) Which delivery option of TempBond was used (ie, automix syringe, tube delivery, or unidose)?

Response: The tube delivery of TempBond was used and completely described in the revised manuscript.

3) - What was your rationale for the low number of thermocycles (ie, 500)? The applied number of cycles corresponds to about 2.6 weeks clinical service (Gale and Darvell 1999).

Response: The number of thermal cycles was mistyped as 500 instead of 5000 cycles. It was corrected in the revised manuscript and respective citation was added.

4) - How did you define the required number of specimens? Was any sample size calculation performed?

Response: Although the sample size had not been statistically calculated, the tests resulted in significant p-values where the effect sizes were moderate to large, indicating that the sample size in each category was sufficient to interpret the results.

5) - Were fabricated crowns randomly assigned to each treatment group?

Response: yes, the fabricated crowns were randomly assigned to each cement group.

6) - How many operators performed the experiments?

Response: As mentioned in the revised manuscript, all color measurements and pull-out tests were performed by a single skilled operator.

7) - Was the operator of the testing machine blinded regarding the treatment group?

Response: Yes, the operator of the testing machine was blinded to the treatment groups.

8) - Please supply LOT numbers of all materials used and provide a table depicting each cements' composition (%).

Response: According to the reviewer's precise comment, a new table was added to mention the LOT number, type and manufacturer of all cements. However, since the composition of provisional resin cements (%) was not clearly reported by the manufacturers, such data could not be included in the table.

9) - Please use uniform spelling/capitalization of all materials throughout the manuscript's main text and figures.

Response: The spelling/capitalization was uniformly revised all over the text and figures.

10) - Please provide the Weibull distribution parameters (Weibull modulus and characteristic strength) of the retentive strength.

Response: The Weibull distribution parameters were not reported in the present study as in the preceding research.

11) - Were all data distributed normally? You only state that normal distribution was tested with Kolmogorov-Smirnov test. However, subsequently both non-parametric tests and parametric tests were used.

Response: In the statistical analysis section, it was stated that Kolmogorov-Smirnov test was used to test normality assumption. The two variables of ΔE00(1) and ΔE00(2) were not normally distributed; hence, non-parametric tests were used for the comparisons. Distribution of retentive strength was normal; thus, parametric tests were employed for the statistical comparisons. The revised manuscript clearly explains the tests used for each variable in.

- Results from Mann–Whitney U test were not corrected for multiple testing.

Response: The results of post-hoc tests (Dunn’s test for nonparametric and Tukey’s test for One-way ANOVA) were displayed in Tables 2 and 5 for each type of restoration. Moreover, the results of Mann-Whitney and t test (p-values) were adjusted based on the number of comparisons. Because the two ceramic restorations were separately compared in each cement (3 cements), the p-values were multiplied by 3 and reported in the revised tables.

Reviewer #2

Introduction

1)(Statement of Relevance) What is the importance of this study for the clinical? You do not think this study is included in the others already done? Which results are comparable? What this study has new?

Response: As mentioned in the Introduction section of the revised manuscript, limited information is available about the effect of provisional resin cements on the final color and retentive strength of implant-supported ceramic restorations. Nor has any study evaluated the effect of TempBond Clear and Implantlink Semi provisional resin cements on the retentive strength and final color of implant-supported all-ceramic restorations before and after thermocycling.

Materials and Methods

2)This section would better communicate to readers if restructured. A flowchart or diagram of the experimental processing would be valuable.

Response: The structure of Materials and Methods section was revised and divided into subsections with definite subheadings.

3)How was the sample calculated? Did the authors perform a power analysis to evaluate if this sample size was appropriate?

Response: Although the sample size had not been statistically calculated, the tests resulted in significant p-values where the effect sizes were moderate to large, indicating that the sample size in each category was sufficient to interpret the results.

4)When mentioning materials or devices: for some of them you don't mention the manufacturer at all, for some you mention only the manufacturer, for some the manufacturer and city, for some you mention the manufacturer and city/ country. Standardized this presentation.

Response: With respect to the reviewer's meticulous suggestion, the manufacturer's detail for each material was standardized as trade name, manufacturer, country.

5)Line 129 - What was the manufacturer's instruction at the time?

Response: The manufacturer's instruction for all the three cements was described in the revised manuscript

6)Please confirm the tensile force at a crosshead speed of 5 mm/min or 0.5 mm/min. Please read this article(https://doi.org/10.1007/s00784-020-03640-7) or <https://doi.org/10.1590/S1678-77572009000600012>.

Response: The tensile force was applied at a crosshead speed of 5 mm/min as mentioned in the manuscript and the respective reference was cited.

Results

7) Improve the resolution quality of all figures and graphs (and a presentation). The font/ language in the figure/caption is different from the text. Please, standardized the size and the font in the figures and charts with the font of the manuscript.

Response: The size and the font in the figures, charts, and body of the manuscript was standardized.

8)Conclusions

The conclusion section should more thoroughly summarize the results, this section is too long and sometime too ambiguous.

Response: The Conclusion section was thoroughly summarized in the revised manuscript.
