# Supplementary material for: The effects of provisional resin cements on the color and retentive strength of all-ceramic restorations cemented on customized zirconia abutments
Source: PLoS One. 2022 Jan 18;17(1):e0262582. doi: 10.1371/journal.pone.0262582 (PMC8765607; doi:10.1371/journal.pone.0262582)
Supplement: Attachment — Submitted filename: Response to Reviewers.docx [file pone.0262582.s004.docx]

Dear Editor,

With deepest appreciations for the great chance of being promoted through PlosOne prestigious journal, attached please find the detailed responses to the reviewers' close concerns. Hope the manuscript now meets all the requirements of publication. Please feel free to contact me for further queries.

Regards, Dr Rashin Giti

Reviewer #1

1)The number of thermocycles was not changed within the discussion section. The manuscript still reads 500 cycles ("500 times between 5° and 55° with a dwell time of 10 seconds was applied").

Response: The number of thermal cycles was corrected in the Discussion section of revised manuscript.

2)Missing sample size calculations increase the risk of bias in laboratory research. Please see the scoring systems for the risk of bias assessment utilized within the following systematic reviews:

Response: Despite missing to calculate the sample size in advance, the statistical tests yienled significant p-values where the effect sizes were moderate to large. It indicated that the sample size in each category was sufficient in this study.

3) For the above-mentioned reason, information regarding the random assignment of the fabricated crowns should be mentioned within the manuscript.

Response: According to the reviewer's wise comment, it was stated in the revised manuscript that the fabricated crowns were randomly assigned to three cement subgroups.

4) For the above-mentioned reason, information regarding the blinded operator should be mentioned within the manuscript.

Response: The operator's blindness to the tested subgroups was clearly mentioned in the revised manuscript.

5) Please extract composition (chemicals and %) from safety data sheets which are provided by the manufacturers.

Response: All of the chemical compositions and % were extracted from safety data sheets and embedded in Table 1.

6)The missing Weibull parameters are a shortcoming of the present study. The authors' reply regarding this topic is unsatisfactory. Please see the following citations for further information regarding the importance of Weibull statistics within the context of dental materials research:

- McCabe and Carrick: A statistical approach to the mechanical testing of dental materials. Dent Mater 1986; 2:139-142.

- Quinn and Quinn: A practical and systematic review of Weibull statistics for reporting strengths of dental materials. Dent Mater 2010; 26:135-147.

Answer: With all due respect to the reviewer's wise comment, the present study missed performing the Weibull statistics.

7) Information regarding the results of the Kolmogorov-Smirnov test is still missing within the text. Also, mentioned statistical test differ between the abstract and the main text. Also, information regarding the meaning of the error bars shown in figures 3 and 4 is missing (eg, stand error, standard deviation). I noticed the revised p-values derived from the Mann-Whitney U tests. Based on the authors' reply, a Bonferroni correction was utilized. However, this information is missing within the text.

Answer: The information regarding the Kolmogorov-Smirnov test was completely added to the Statistical analysis section of revised manuscript, also the total statistical test was added to the Abstract section of revised manuscript. Besides, the legends of Figures 3 and 4 state that the means and standard deviations of each variable were shown in the figures.

As presented in the Statistical Analysis section, all the statistical teste used in the presented study are as follows: Normal distribution was tested with the Kolmogorov-Smirnov test. Kruskal-Wallis, Dunn’s post-hoc, and Mann-Whitney non-parametric tests were used to compare ΔE00(1) and ΔE00(2) among the study groups and subgroups, because the data for these two variables (ΔE00(1) and ΔE00(2)) were not normal. After the normality test showed that the data for retentive strength was normal, the mean retentive strength was analyzed with two-way ANOVA followed by one-way ANOVA and Tukey's HSD post hoc test to determine statistically significant differences among the three employed cements. T-test was used to compare the retention of ceramic crowns as a function of the type of cement. P<0.05 was considered to be statistically significant in all tests.
