# Supplementary material for: The effects of provisional resin cements on the color and retentive strength of all-ceramic restorations cemented on customized zirconia abutments
Source: PLoS One. 2022 Jan 18;17(1):e0262582. doi: 10.1371/journal.pone.0262582 (PMC8765607; doi:10.1371/journal.pone.0262582)
Supplement: Attachment — Submitted filename: Response to Reviewers.docx [file pone.0262582.s005.docx]

Dear Editor,

We would like to appreciate your meticulous review and comments. The manuscript was revised accordingly and a point-by-point response comes as following. Hope the manuscript now meets all the publishing requirements.

Regards, Dr Rashin Giti

Reviewer #1

1. Information regarding my previous 7th comment is still missing ("Based on the authors' reply, a Bonferroni correction was utilized. However, this information is missing within the text."). Please add information within the statistical analysis section, if and how p-values derived from Mann-Whitney U and t tests were adjusted for multiple testing (eg, Bonferroni correction, Bonferroni-Holm correction).

Response: Details of the employed Bonferroni correction was added to the statistical analysis section of revised manuscript and highlighted.

2. Table 2: The Lilliefors correction is related to the Kolmogorov-Smirnov test for normality. Therefore, the footnote "* Lilliefors Significance Correction is P<0.05" is misleading.

Response: This part was deleted as suggested by the reviewer.

3. Table 2: "0.999a©b" should be written as "0.999ab©".

Response: It was corrected as recommended.

4. Tables 5: The footnote "Significance correction is P<0.05" not required.

Response: It was omitted according to reviewer's comment.

5. Tables 3 and 6: Again, the footnote "Significance correction is P<0.05" not required if information regarding the level of significance and adjustment for multiple testing is given within the statistical analysis section.

Response: It was omitted as suggested.

6. Please write p-values as "<.001" instead of ".000".he footnote "* Lilliefors Significance Correction is P<0.05" is misleading.

Response: The P-values were corrected according to the reviewer's meticulous comment.
